# Apoptotic Potential of Iloneoside from *Gongronema latifolium* Benth against Prostate Cancer Cells Using In Vitro and In Silico Approach

**DOI:** 10.1007/s12013-024-01507-2

**Published:** 2024-09-20

**Authors:** Gideon A. Gyebi, Saheed O. Afolabi, Oludare M. Ogunyemi, Ibrahim M. Ibrahim, Olufunke E. Olorundare, Joseph O. Adebayo, Mamoru Koketsu

**Affiliations:** 1 Department of Biotechnology and Food Science, Faculty of Applied Sciences, Durban University of Technology, Durban, South Africa; 2 Natural Products and Structural (Bio-Chem)-informatics Research Laboratory (NpsBC-RL), Department of Biochemistry, Faculty of Science and Technology, Bingham University, Karu, Nigeria; 3 Biomolecular Modeling and Nutraceuticals Laboratory, Nutritional and Industrial Biochemistry Research Unit, Department of Biochemistry, College of Medicine, University of Ibadan, Ibadan, Nigeria; 4 Faculty of Basic Medical Sciences, Department of Pharmacology and Therapeutics, University of Ilorin, Ilorin, Nigeria; 5 Department of Biophysics, Faculty of Sciences, Cairo University, Giza, Egypt; 6 Department of Biochemistry, Faculty of Life Sciences, University of Ilorin, Ilorin, Nigeria; 7 Faculty of Engineering, Department of Chemistry and Biomolecular Science, Gifu University, Gifu, Japan

**Keywords:** *Gongronema latilolium*, Prostate cancer, Apoptosis, Pregnanes, Iloneoside, Molecular docking

## Abstract

Prostate cancer is a major cause of cancer-related mortality in men worldwide. The anti-proliferative activity of *Gongronema latifolium* leaf extracts on some cancer cells has been reported. Herein, we investigated the growth inhibitory effect of the *Gongronema latilolium* leaf methanol extract and isolated pregnane (iloneoside) against prostate cancer cell lines using the MTT cell proliferation assay, apoptosis quantification, cell cycle analysis using flow cytometry and computational analysis molecular docking, molecular dynamics simulation (MDs), binding free energy computation and cluster analysis. In addition, UPLC-ESI-TOFMS chemical fingerprinting of previously isolated compounds was performed. The extract inhibited the growth of the cell lines with an IC_50_ of 49.3 µg/ml and 28.4 µg/ml for 24 h and 48 h, respectively, for PC3; and 43.7 µg/ml and 22.3 µg/ml for 24 h and 48 h, respectively, for DU145. Iloneoside demonstrated low inhibitory activities against PC3 and DU145 (IC_50_ > 80 μM). Apoptotic quantification and cell cycle analysis further showed that iloneoside induced apoptosis in a few cells at a dose of 200 uM. The ensemble-based molecular docking of the iloneoside to BCL-XL and BCL-2 proteins, and docking to MCL-1, BCL-A1 and BFL-1 proteins, respectively, presented binding energies of −7.22 ± 0.5, −8.12 ± 0.55, −7.1, −7.2 and −6.3 kcal/mol, while the MM/PBSA binding free energy was −25.72 ± 7.22 and −27.76 ± 11.32 kcal/mol for BCL-XL and BCL-2 proteins. Furthermore, iloneoside was stable during the 100 ns MDs analysis, while the clustering of the MDs trajectories showed that the interactions were strongly preserved. Iloneoside, in part, or in synergy with other constituents, may be responsible for the antiproliferative activities of the leaf, subject to further investigation.

## Intoduction

Prostate cancer is a major cause of cancer-related mortality in men worldwide, affecting between 9% and 11% of men [1–3]. Prostate cancer arises from unchecked cell division, which leads to aberrant cell proliferation and metastases to other body areas [4]. Prostate cancer management has made significant progress in the past 20 years, but the disease continues to represent a public health risk and necessitates the development of novel chemopreventive and chemotherapeutic drugs in addition to the hormonal drugs and taxane chemotherapy that are now on the market [5]. Inducing apoptosis in cancer cells has long been a desirable therapeutic target for cancer treatment [6]. Apoptosis, also referred to as the ‘mitochondrial’ apoptosis pathway, is a highly controlled and regulated process of cell death that falls into two main categories: intrinsic and extrinsic [7]. Conventional and well-researched treatment approaches for prostate cancer, such as chemotherapy and androgen restriction therapy, cause cellular stress, which can then trigger the intrinsic apoptotic pathway. The BCL-2 protein family, which consists of pro- and anti-apoptotic proteins that interact on the mitochondrial outer membrane and share one to four BCL-2 homology (BH) domains (BH1, BH2, BH3, BH4), closely modulates the ‘mitochondrial’ apoptosis pathway [8]. Pro-apoptotic BCL-2 members can be either BH3-only sensitisers (Bcl-2-associated death promoter (BAD), NOXA, Harakiri (HRK), Bcl-2 interacting killer (BIK), Bcl-2-modifying factor (BMF), or pore-forming effectors (BAX, BAK). BH3-only activators include Bcl-2-like protein 11, BID and PUMA. Proapoptotic proteins are known to be counteracted by anti-apoptotic/pro-survival BCL-2 proteins, such as B-cell lymphoma 2 (BCL-2), Myeloid-cell leukaemia 1 (MCL-1) and B-cell lymphoma-extra large (Bcl-XL) [9]. Therefore, the interplay between pro- and anti-apoptotic BCL-2 family proteins is essential in determining cell fate decisions and it is appealing to manipulate this interplay in cancer therapies to exceed the apoptotic threshold [10]. There are various initiatives underway to target BCL-XL and MCL-1 since solid tumours, including prostate cancer, seem to depend more on these proteins for cell survival [11].

Agents that target the intrinsic apoptotic pathway have been developed thanks to recent developments in medicinal chemistry. Early phase trials are now being conducted to test these inhibitory drugs, with BH3 mimetics being the most clinically progressed agent [11]. The BH3 mimetics are a class of small compounds that are engineered to mimic the binding of BH3-only proteins to the hydrophobic groove on anti-apoptotic BCL-2 proteins. This allows BH3-only proteins to be released to exert their pro-apoptotic action on BAX/BAK and/or spontaneously activate BAX/BAK. Venetoclax is an FDA-approved BCL-2 inhibitor that specifically targets the intrinsic machinery involved in apoptosis. All anti-apoptotic BCL-2 family proteins were suppressed by the first generation of ‘pan-BH3 mimetics,’ including AT-101 and obatoclax, but their clinical development was stopped by off-target dose-limiting toxicities [12]. Different anti-apoptotic proteins have different affinities for the BH3 mimetics that mimic endogenous BH3-only proteins. Among these, ABT-737 was created to specifically inhibit BCL-2, BCL-XL and BCL-W. It has shown encouraging results against mouse xenograft models of lymphoma and small-cell lung cancer [13]. But because of its undesirable pharmacokinetic characteristics, navitoclax (ABT-263), an orally accessible substitute, was created [14].

Given that between 43 and 80 percent of prostate cancer patients are receiving alternative therapy that involves dietary modification, food plants and their bioactive components present a promising bioresource for chemopreventive and/or therapeutic nutraceuticals in the modern day [15–17]. Strong evidence suggests that when evaluated in vitro and/or in vivo, nutraceuticals and plant-derived extracts have shown notable anti-prostate cancer activity [17, 18], especially, when they can selectively interfere with cellular pathways involved in prostate [19]. There have been reports of anticancer properties for the food plant *G. latifolium* Benth (Asclepiadaceae), which is eaten as a green leafy vegetable. *G. latifolium* and a number of other plants have been linked to cancer chemoprevention by Ohiagu, Chikezie [20]. Extract derived from *Morus nigra* was shown to exhibit antiproliferative and apoptotic effect on human prostate cancer cells [18]. *G. latifolium* leaf extracts were reported by Gyebi et al. [21] to exhibit strong inhibitory activity against leukaemia cells in vitro. Additionatlly, in vitro studies by Iweala et al. [22] revealed that G. latifolium leaf extract had a potent inhibitory effect against human lung cancer and human breast adenocarcinoma.

Plant-derived pregnane compounds have been shown to exhibit cytotoxic activities in different cancer cells revealing their potentials in chemoprevention and cancer therapy. Two pregnanes isolated from the leaves of *Adenium obesum*, were demonstrated to exhibit a cytotoxic activity against murine leukaemia P388/S cells [19]. Three novels steroidal pregnane structures and eleven new C21 steroidal glycosides were identified in another investigation as being extracted from the vines and leaves of *Chonemorpha megacalyx* Pierre. These compounds demonstrated notable cytotoxicity against a number of cancer cell lines, with IC_50_ values ranging from 2.0 to 3.6 μM [23]. In vitro studies with three breast cell lines (MCF-7, MCF-10A and ZR-75-1) showed the inhibitory potential of several pregnanes [24]. A study also revealed that a new pregnane glycosides alongside several pregnane isolated from 80% methanolic extract of the leaves of *G. latifolium* Benth exhibited growth inhibitory activity against human leukaemia HL-60 cells and gave pro-apoptotic response [21]. Although there are studies on the the inhibitory effect of the extract on other forms of cancer cells, there is no report on the prostate cancer, furthermore the constituent responsible has not been elucidate. Therefore, the current study focuses on the apoptotic investigation of iloneoside derived from *G. latifolium* against prostate cancer cells using in vitro and in silico approaches.

## Matetials and Methods

### Materials

#### Plant materials

*Gongronema latifolia* leaves from Okuku in Osun state in November 2017, South-western Nigeria was authenticated by Mr. Bolu Ajayi a Botanist/Curator at the Herbarium of the Department of Plant Biology, University of Ilorin, Ilorin, Nigeria, where a voucher sample (voucher number: UILH/002/1176) was deposited.

#### Preparation extract and solvent solvent partitioning

Our earlier research described the procedures for extract extraction, solvent partitioning and iloneoside structural elucidation [21].

#### Cell lines

The American Type Culture Collection (ATCC) provided PC3 and DU145 prostate cancer cells, which were then cultured in RPMI 1640 (Life Technologies, NY) supplemented with 10% FBS and 1% penicillin/streptomycin, with 5% CO_2_ at 37 °C. Cells were treated with a solution of the plant extract and iloneoside in DMSO.

After confluency of ~70%, cells were treated with extract and iloneoside for 24 h in full cell media. The final DMSO concentration utilised in each treatment was less than 0.1% (v/v).

#### UPLC-ESITOFMS conditions

The method was adopted from from Afolabi et al. [25]. An aliquot (5 μl) of the samples—which included the isolates and the ethyl acetate fraction—were then injected into the UPLC after being dissolved in DMSO/H2O (1/1) at a concentration of 5 mg/ml and filtered through a 0.45 μm membrane filter (ADVANTEC®, Japan). Analyses were recorded using a UPLC BEH C18 analytical column (1.7 μm, 2.1 × 100 mm) and the Waters UPLC system (Aquity UPLC XevoQTof). Solvent A (1% v/v AcOH in distilled water) and Solvent B (acetonitrile) made up the mobile phase. Using a liner gradient system, the following ratios were used: 90% solvent A to 70% solvent A and 10% solvent B to 30% solvent B for 0–30 min; this ratio was then held at 70% A (30% B) for 5 min; in 35–45 min, 70% solvent A to 50% solvent A and 30% solvent B to 50% solvent B. We kept an eye on the column eluate [26].

### In Vitro Assay

#### Cytotoxicity Assay

Using the 3-(4,5-dimethylthiazol-2-yl)-2,5-diphenyl tetrazolium bromide (MTT) test, the impact of iloneoside and *G. latifolium* leaf extract on the survival of PC3 and DU145 cell lines was investigated. In 96-well microtiter plates, the cells were plated (1 × 10^4^ cells per well) in 200 µl of complete culture media. Cells were treated with different doses of 0–140 μg/ml for the extract and 0–120 μM for iloneoside dissolved in DMSO 0.1% (v/v) and incubated for 2 h at 37 °C and 5% CO_2_ in a humidified incubator. Following this, 100 μl of MTT (5 mg/ml:1XPBS) was added. After that, the plates were centrifuged for 5 min at 4 °C (1800 × *g*). A microtitre plate reader was used to record the absorbance at 540 nm. The percentage of cell viability was used to calculate the cytotoxic effect of extract and iloneoside on the cell lines.

#### Cell cycle analysis and apoptotic cell quantification using flow cytometry

Methods described by Shabbir et al. [27]. PC3 cells were trypsinized and fixed in 1% paraformaldehyde after being treated with iloneoside 200 μM for 24 h in full media. 1X PBS for an hour, followed by two cold PBS washes and suspension in 70% ethanol that has been cooled. The following day, the cells were centrifuged for 5 min at 1000 rpm and the resulting pellet was twice cleaned of ethanol using cold PBS. Following the manufacturer’s instructions, the Apo-Direct Kit (BD Pharmagen, CA) was used to label the cells with FITC and propidium. An FACScan was used for the analysis (Becton Dickinson, NJ). About 10,000 events per sample were collected, and the DNA histograms were analysed with ModFitLT software [25].

### Statistical analysis

Using IBM SPSS Statistics for Windows, version 23.0 (IBM Corp., Armonk, N.Y., USA), data were analysed for statistical significance using one-way analysis of variance and Duncan’s post hoc multiple comparisons. A difference was deemed significant at p < 0.05. The Windows programme GraphPad Prism 6 (GraphPad Software, California, USA) was used to make the graphs.

### In Silico Assay

#### Instrumentation

Molecular dockings calculations were performed on HP ZBook G5 Intel(R) Core (TM) i7-7700HQ CPU @ 2.80 GHz 2.81 GHz and 32.0 GB GB ram, while MMPBSA and MDS computation was performed on a 2x Intel Xeon E5-2680 v3 (24 threads) processors situated in Bibliotheca Alexandria HPC facility, Alexandria, Egypt.

#### Retrieval and preparation of proteins

The methods employed in this study has been previously described in our articles [28, 29] The anti-apoptotic BCL-2 proteins (APB2P) crystal structures that were needed for the docking investigations were obtained from the Protein Databank (http://www.rcsb.org) with their various PDB identification codes (BCL-2 (4lvt) [30]; BCL-XL (3zlr) [31]; MCL-1 (5fdr) [32]; BFL-1 (5uul) [33] and BCL-A1 (5whi) [34]. Using MGL-AutoDockTools (ADT, v1.5.6), the anti-apoptotic proteins were processed by eliminating the water molecules and existing ligands, adding missing hydrogen atoms in accordance with the amino acid protonation state at pH 7.0, and adding Kollamn charges as partial atomic charges [35]. After that, polar hydrogens were added to each protein and non-polar hydrogens were combined. Each protein underwent the same procedure, it was thereafter saved for molecular docking in a dockable pdbqt format.

#### Ligand preparation

The reference inhibitors’ Structural Data Format (SDF) structures were downloaed from www.pubchem.ncbi.nlm.nih.gov (PubChem database) and transformed using Open Babel into mol2 chemical format. [36], whereas ChemDraw 19.0 was used to sketch and convert iloneoside to the mol2 format. By using the same procedure as for protein creation, nonpolar hydrogen molecules were combined with the carbons and polar hydrogen charges of the Gasteiger type were assigned. Using Autodock tools, the ligand and protein molecules were further transformed to dockable pdbqt format.

#### Active site targeted and optimised molecular docking

An ensemble-based molecular docking analysis was conducted, wherein the iloneoside and reference compounds were docked to multiple conformers derived from the cluster analysis of the molecular dynamics trajectories of the unbound BCL-2 and BCL-XL proteins. Meanwhile, a standard molecular docking analysis was utilised to dock the iloneoside to the active site of MCL-1, BFL-1 and BCL-A1.

The docking was performed using AutoDock Vina that have been incorporated in the PyRx 0.8 software (Trott and Olson, 2010). The test substances were imported, and PyRx 0.8’s OpenBabel was used to minimise energy consumption. Conjugate gradient descent was employed as the optimisation approach with energy minimisation and the Universal Force Field (UFF) as the energy minimisation parameter.

(O’Boyle et al. [36]). Docking studies were conducted using the binding sites region of the target proteins, which was determined by a grid box size and centred (Table 1).

**Table 1:** The coordinates for the binding site coordinates of target proteins

Protein PDB ID	center_x	center_y	center_z	Size_x	Size_y	Size_z
4LVT	24.504	32.51	28.22	18.67	23.20	23.367
3ZLR	4.77	24.02	−47.40	22.19	20.72	18.67
5FDR	7.60	3.51	17.86	25.0	28.68	20.72
5UUL	−9.21	4.25	−13.59	25.0	18.46	13.63
5WHI	−10.31	10.06	−15.60	18.53	25.0	30.81

Every other parameter was left at its default setting. To decrease energy use, the test chemicals were imported and OpenBabel from PyRx 0.8 was utilised. The optimisation method used was conjugate gradient descent, with the Universal Force Field (UFF) serving as the energy minimisation parameter and energy minimisation as the goal.

The cluster analysis of the MD trajectory of BCL-2 and BCL-XL was performed using the TTClust V 4.9.0 [37] following the methods reported in our previous studies [38].

Using the TTClust Python package, which uses the elbow approach to calculate the ideal number of clusters and then generates a representative frame for each cluster, each system was automatically clustered. From each cluster, a representative conformation was chosen to be used in the detailed docking experiment.

#### Molecular dynamics simulation

MDs using Nanoscale Molecular Dynamics (NAMD) V2.13 [39] was used to study the dynamics of 3ZLR (unbound), and its complex with Iloneoside and 4LVT (unbound) and its complex with Ilneoside. Using the CHARMM-GUI webserver, the required files were prepared before initiating MDS [40, 41] (2,3). All systems were neutralised using NaCl ions at the physiological concentration of 0.154 M after being solvated in the TIP3P water model. Using CHARMM 36 force field and a conjugate gradient technique, each system was minimised for 10,000 steps in the constant number of atoms, constant volume and constant temperature ensemble (NVT).

Subsequently, equilibration was initiated in an NPT (number of atoms constant, pressure constant and temperature constant) ensemble for one nanosecond. Pressure was set to 1 atm and maintained using a Nose-Hoover langevin piston, while temperature was set to 310 K and maintained via langevin dynamics. Lastly, each system ran in the NVT ensemble for 100 ns. Before beginning the analysis, Periodic Boundary Conditions (PBC) were set during the simulation and then removed. The time step was set to two femtoseconds, and trajectories were stored every 0.1 ns. The trajectories were analysed using VMD scripts, which computed the H-bonds, Radius of Gyration (RoG), Surface Accessible Surface Area (SASA), Root Mean Square Deviation (RMSD) and Root Mean Square Fluctuation (RMSF) [42].

#### Binding free energy calculations

The Molecular Mechanics Generalised Born Surface Area (MMGBSA) function, which can be found in MMPBSA.py of the Ambertools 17 package, was used to calculate the binding free energy. The saltcon variable was set to 0154 M, and the generalised birth method (igb) was set to 5. Furthermore, by breaking down the free energy, the contribution of each amino acid to the binding was determined. [43, 44].

#### Clustering of molecular dynamic trajectory

Each trajectory was clustered using TTClust V 4.9.0 [37] by utilising the elbow approach to determine the ideal number of clusters. The number and nature of interactions between the compounds and the protein were ascertained using the Protein-Ligand Interaction Profiler (PLIP) [45]. Amino acids numbers in all of the analyses starts from number 1 until the end of the sequence without any gaps.

## Results

### Chromatographic Profiling of Iloneoside Isolated from *Gongronema latifolium*

**Fig. 1:**
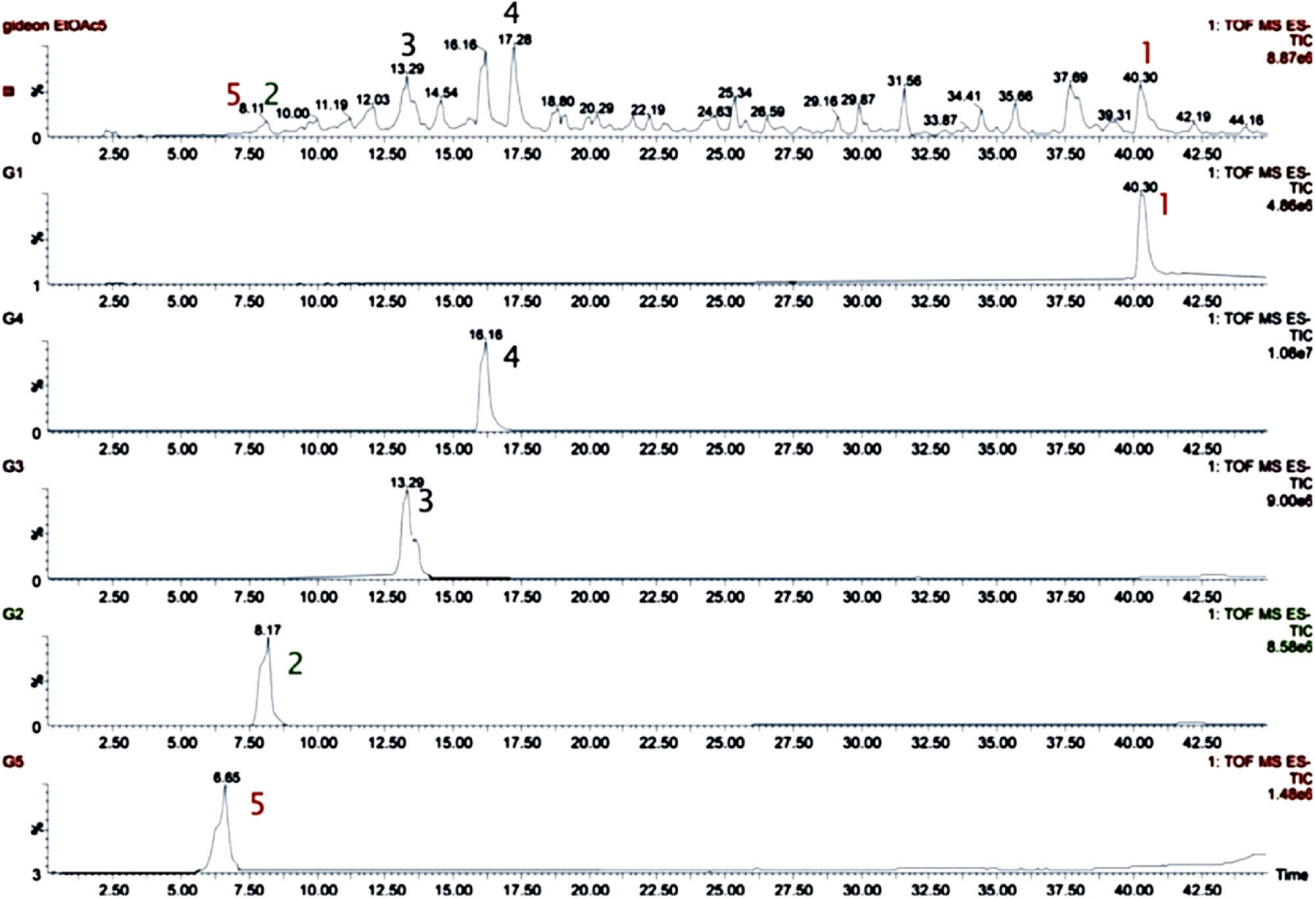
UPLC analysis of Isolated compounds in the EtOAc fraction of methanol extract of *G. latifolium* leaves

The chromatographic profile of compounds iloneoside in the EtOAc fraction of *G. latifolium* from which iloneoside was isolated was investigated using UPLC-ESI-TOFMS. The peaks for iloneoside were identified at a retention time of 40.30 (Fig. 1). Iloneoside was one of the major constituents of the EtOAc fraction.

### Cytotoxicity Test

**Fig. 2:**
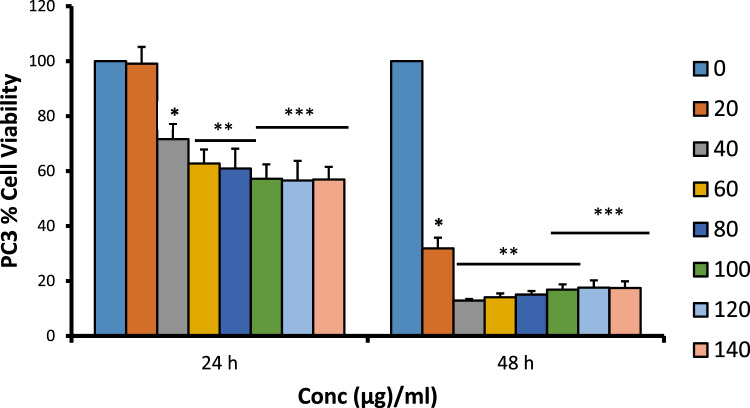
Cytotoxic effects of ethanol extract of *G. latifolium* leaves on prostate cancer cell line PC3 (means ± SEMs, *n* = 10). Values for each time duration with different superscripts are significantly different at p < 0.05

**Fig. 3:**
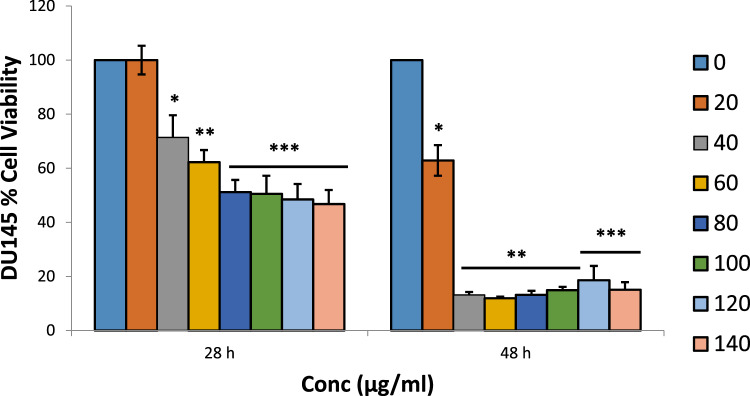
Cytotoxic effects of ethanol extract of *G. latifolium* leaves on DU 145-cell line (means ± SEMs, *n* = 10). Values for each time duration with different superscript are significantly different at p < 0.05

MTT assays against androgen-independent prostate cancer cell lines PC3 and DU-145 showed that the ethanol extract of Gongronema *l*. inhibited the growth of the cell lines with an IC_50_ of 49.3 µg/ml and 28.4 µg/ml for 24 h and 48 h, respectively, for PC3 and 43.7 µg/ml and 22.3 µg/ml for 24 h and 48 h, respectively, for DU145 (Figs. 2 and 3).

**Fig. 4:**
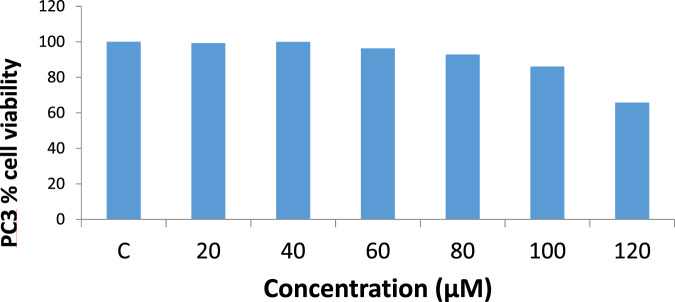
Cytotoxic effects of iloneoside on PC3 cell lines

**Fig. 5:**
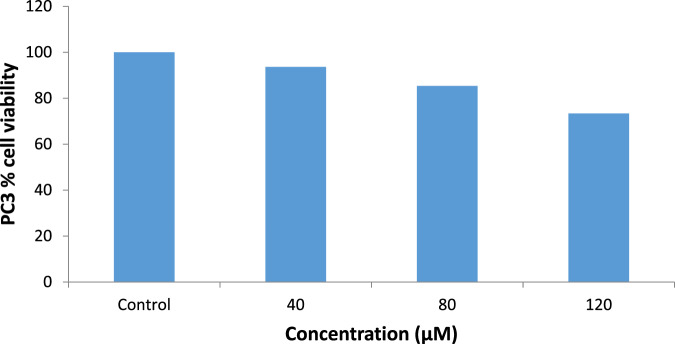
Cytotoxic effects of iloneoside on DU145 cell lines

A further antiproliferative study of iloneoside using an MTT assay against PC3 and DU-145 cell lines showed low growth inhibition with an IC_50_ > 80 μM (Figs. 4 and 5).

### Apoptosis Quantification and Cell Cycle Analysis Using Flow Cytometry

**Fig. 6:**
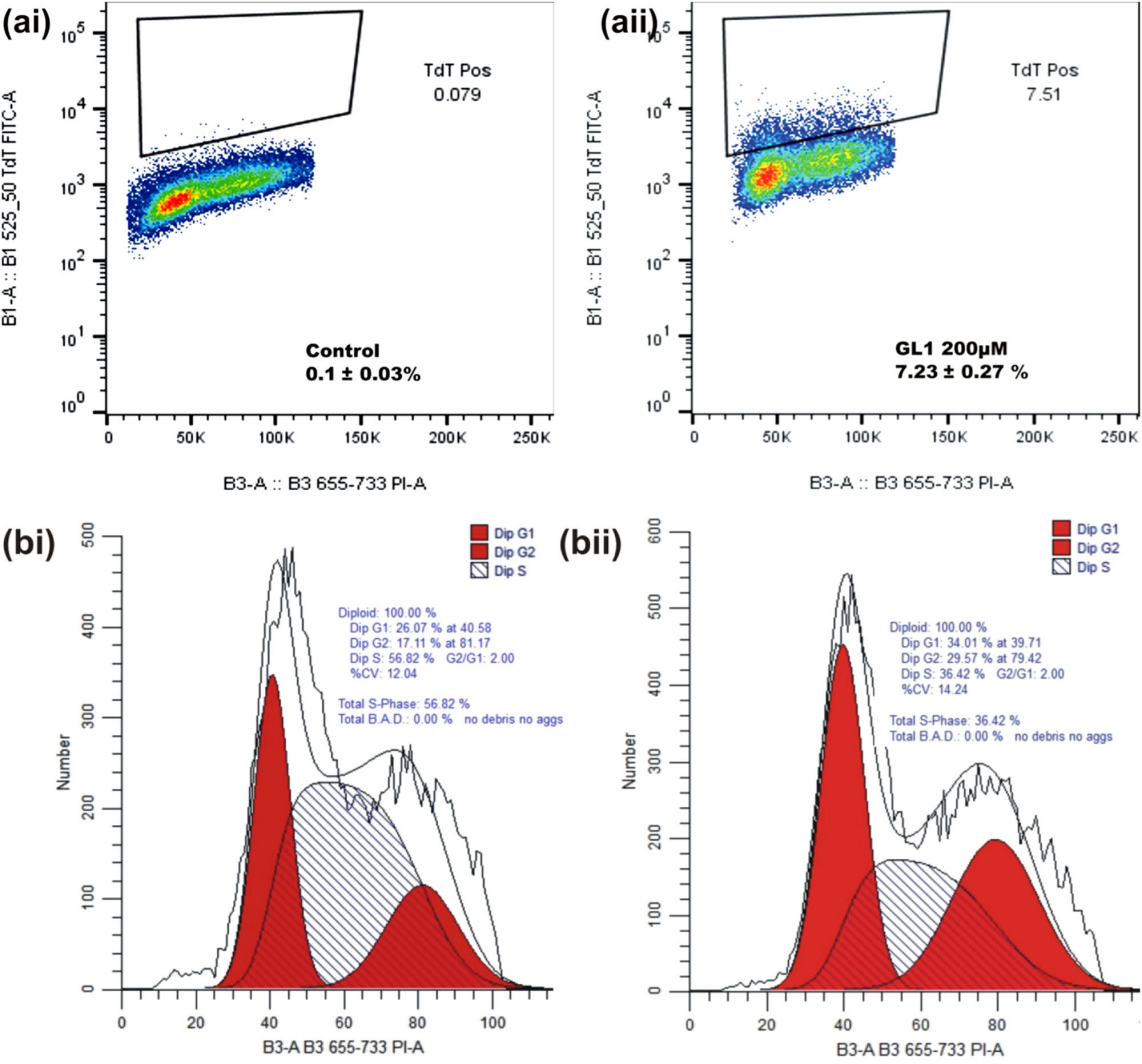
**a** Percentage quantification of FITC-labelled apoptotic PC3 cells (**ai**) untreated cells (**aii**) treated with iloneoside (200 µM) for 48 h (**b**) Cell cycle analysis of PC3 cells, showing the distributions of cells at the G0/G1 and G2M checkpoints (**bi**) untreated cells (**bii**) treated with iloneoside (200 µM) for 48 h. (Mean ± SEM n = 3)

To comprehend the mechanism of observed cell death, apoptosis detection and quantification were evaluated. Iloneoside (200 µM) treated cells showed a 7.23 ± 0.27% increase in the fraction of apoptotic DNA, as depicted in Fig. 6. The cell cycle profile of iloneoside*-*treated PC3 cells was elucidated using flow cytometric analysis, and the effect of treatment on the cell cycle distribution was ascertained. The G1, G2 and S phase cell cycle distributions for the untreated PC3 cells are 23.07%, 17.11% and 56.82%, while those of the iloneoside treated PC3 cells are 34.01%, 29.57%, and 36.42%. Iloneoside-treated cells showed an increase in cell population at the G_1_ and G_2_ phases but a reduction in the S phase of the cell cycle.

### In silico Interaction of Compounds with Selected Cancer Target proteins

Iloneoside and reference compounds were docked to the representative conformers that were generated from the four and three clusters of the MD trajectories of Bcl-2 and BCL-XL, respectively, for a thorough molecular docking investigation. In the supplemental data, the thermodynamic parameters (SASA, RMSD, RoG and RMSF H-bond numbers) of the unbound, the snap short of the representative structure and the cluster frame numbers are represented. The best representative structure with the best docked pose and minimal binding energy was selected for interactive analysis. Based on the observed maximum binding energy with iloneoside, a representative conformer from cluster 1 was chosen as the best representative conformation of Bcl-2 and BCL-XL. The means and standard errors of mean for the binding affinities from the ensemble-based docking analysis of iloneoside and the reference compounds (navitoclax, WEHI-539 and gambogic acid) against BCL-2, and BCL-XL and the binding affinities of iloneoside and the reference compounds to MCL-1, BCL-A1 and BFL-1 are shown in Table 2.

**Table 2:** Binding affinity of standards (S1, S2 and S3) and iloneoside to the BCL-2, BCL-XL, MCL-1, BFL-1 and BCL-A1 anti-apoptotic proteins

S.No	compounds	Binding affinity (kcal/mol)				
		Bcl-2	BCL-XL	Mcl-1	Bfl-1	Bcl A1
S1	Gambogic Acid	−7.22 ± 0.54	−7.70 ± 0.23	−9.9	−7.6	−8.3
S2	Navitoclax	−8.25 ± 1.4	−8.97 ± 0.49	−10.8	−10.4	−9.4
S3	WEHI_539	−7.61 ± 0.45	−9.12 ± 0.30	−8.6	−8.4	−8.4
	Iloneoside	−7.22 ± 0.5	−8.12 ± 0.55	−7.1	−7.2	−6.3

#### Amino acid interaction of selected bioactive compounds with anti-apoptotic proteins

**Fig. 7:**
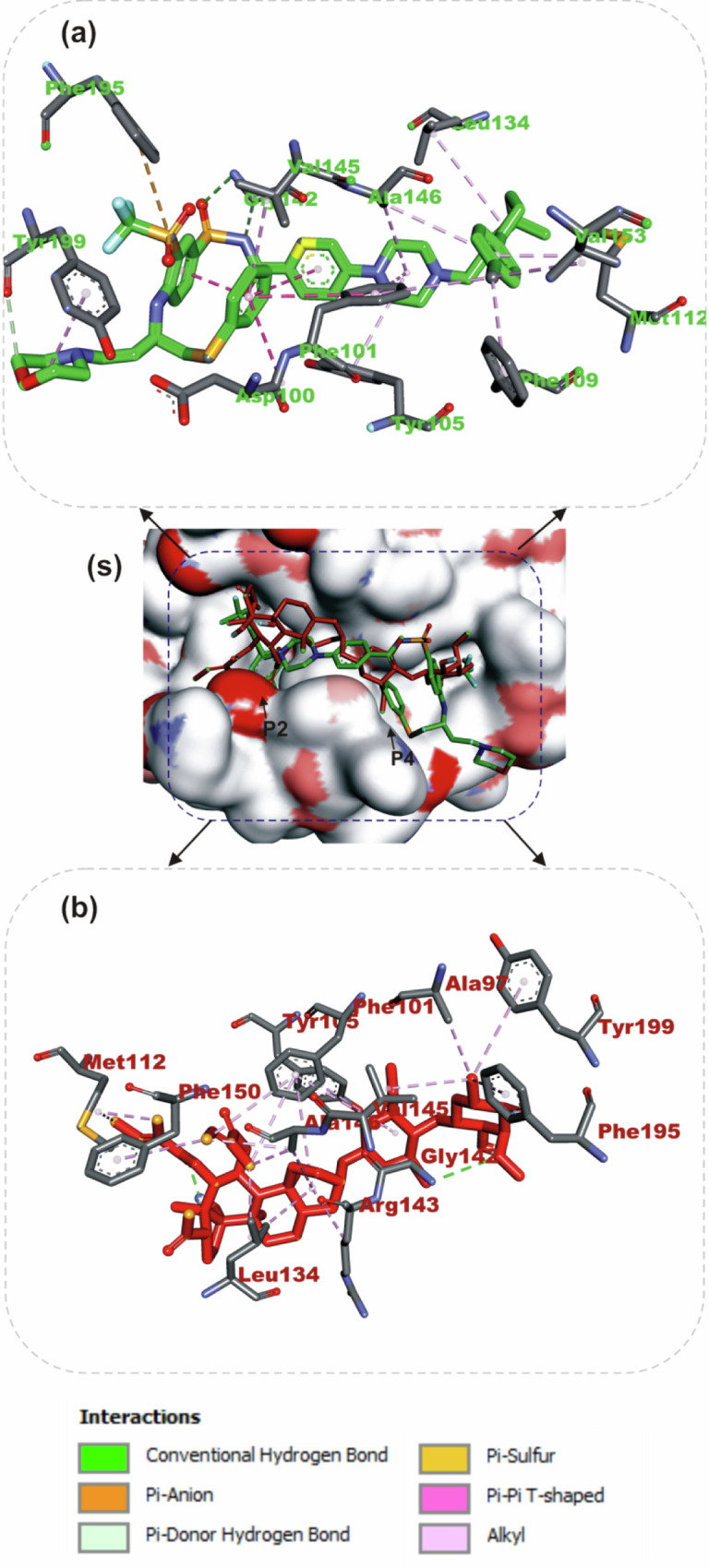
The binding mode details are shown in (**s**) the solvent-accessible surface view and (**a**, **b**) the interaction view of the ligands in the Bcl-2 BH3 binding pocket. Colours indicate stick representations of the ligands. **a** Green: referencer inhibitors, such as navitoclax; **b** Red: iloneoside

**Fig. 8:**
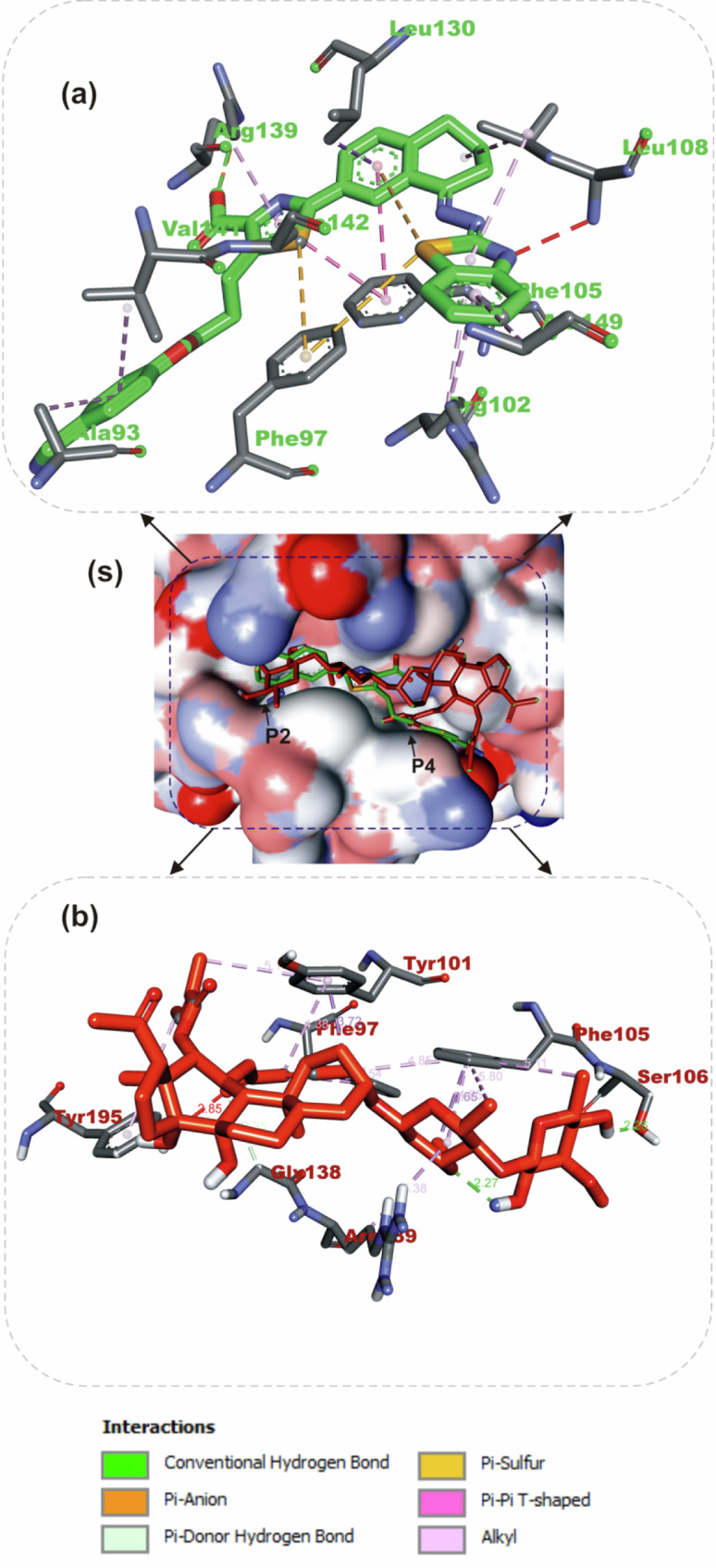
The binding mode details are shown in (**s**) the solvent-accessible surface view and (**a**, **b**) the interaction view of the ligands in the Bcl-XL BH3 binding pocket. Colours are used to represent the ligands as sticks: **a** green for WEHI_539 (referencer inhibitors); **b** red for iloneoside

**Fig. 9:**
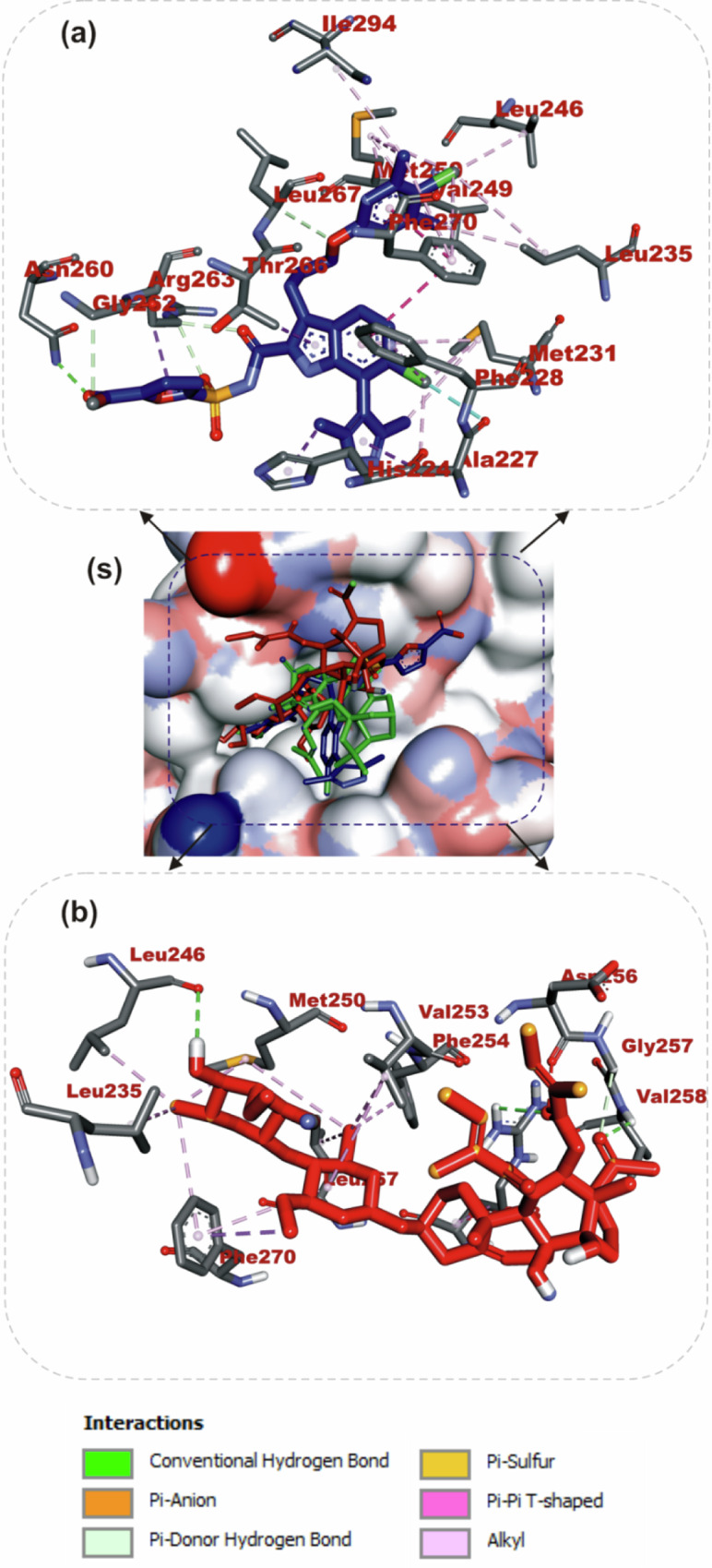
Specifics of the binding mode (**s**): Liquid-accessible surface view; (**a**, **b**): Ligand interaction view in Mcl-1’s BH3 binding pocket. The colours (**a**) green indicate stick representations of the ligands. 2. Referencer inhibitors, indole-acylsulfonamide (**b**) red: iloneoside

**Fig. 10:**
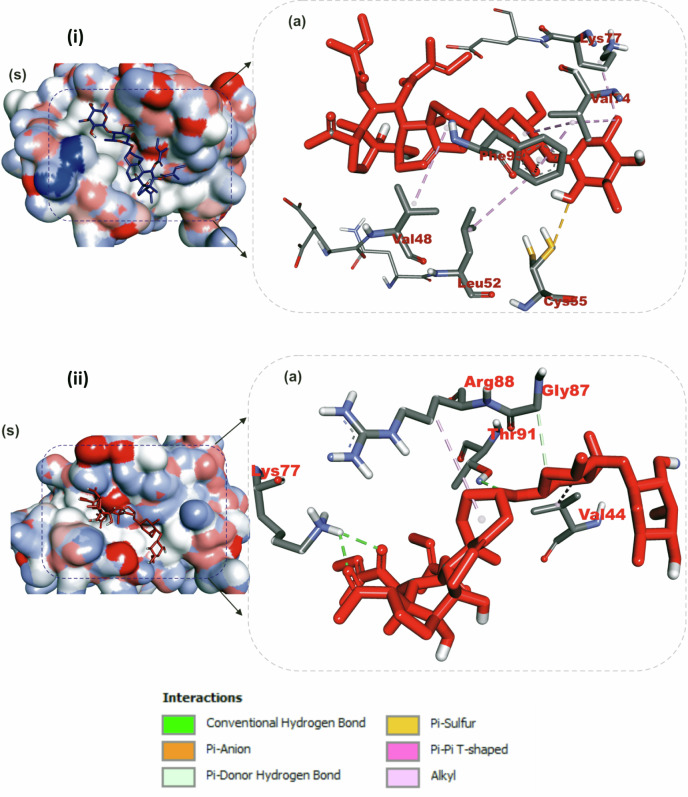
Details about the binding mode Solvent-accessible surface image (**a**); interaction view of ilneoside in (**i**) Bfl-1 and (**ii**) BCL-A1 BH3 binding pocket (**a**). Stick representations of the iloneoside are shown in red colours

The interactions of iloneoside and the reference inhibitors with the amino acid residues of five APB2Ps are represented in Table 3. The interacted residues of the APB2Ps with respective ligand groups were predominantly hydrophobic (Table 2), and few H-bonds below 3.40 Å were observed. The revalidation analysis of the docking pattern of the native ligand (navitoclax) that was co-crystalized with Bcl-2 protein showed that navitoclax was docked into the BH3 binding groove of Bcl-2. Navitoclax stretched into the P2 and P4 binding pockets of the groove. In a similar binding mode as navitoclax, the best docked pose of iloneoside was accommodated in the BH3 binding groove of Bcl-2 (Fig. 7a). Like the native ligand, the 17β-marsdenin steroid aglycon of iloneoside was aligned in the P2 subunit, and the glycoside unit was stretched towards the P4 pocket of the Bcl-2 (Fig. 7b). The interaction was primarily stabilised by one hydrogen bond and several alkyl contacts. The 3-O-methyl group attached to the allopyranosyl interacted with a hydrogen bond to Gly142. The two O-tigloyl moieties attached to carbons 11 and 12 of aglycon made contact with Met112, Leu134, Phe101, Ala146 and Phe150 of Bcl-2. The A ring made Pi-Alkyl contact with Ala146 and Arg143, while the 5-hydroxyl group on the allopyranosyl sugar formed four alkyl interactions with Val145, Ala97, Phe195, and Tyr199 of Bcl-2 (Fig. 7b). Iloneoside was also docked into the BH3 binding groove of BCL-XL in the same orientation as the native ligand WEHI_539, showing distinctive interaction with PHE105 in the P2 binding pocket of BCL-XL (Fig. 8a). The two tigloyl units were directed into the hydrophobic cleft of the binding groove and stabilised with Pi-Sigma and alkyl interactions to Tyr101 and Phe97, respectively, while the steride basic skeleton was arranged on the outside of the binding regions of BCL-XL (Fig. 8b). The carbonyl group of the tigloyl group attached to carbon 11 of the aglycon formed a hydrogen bond with Gly138, while the 4-hydroxyl group of the allopyranosyl sugar formed a hydrogen bond with Ser106. The 3-O-methyl group of the oleandropyranosyl also made a Pi-Sigma contact with Phe105 of BCL-XL (Fig. 8b). The best conformation with the minimal binding energies of gambogic acid, a known inhibitor of Mcl-2 and iloneoside were docked in the same binding hot spot of 2-indole-acylsulfonamide, the native ligand that is co-crystallised with Mcl-1 (Fig. 9S). The two ligands interacted with residue Arg263 in an H-bond (Fig. 9a, b). The ketone carbonyl formed two hydrogen bonds with Gly257 and Val258, the carbonyl oxygen of the carbon 12-O-tigloyl group interacted through a hydrogen bond with Arg263 and the 4-hydroxyl group of the allopyranosyl formed the last hydrogen bond with Leu246 of Mcl-1 (Fig. 9b). The C-6 methyl group of allopyranosyl sugar made four alkyl contacts with Leu246, Leu235, Met250 and Phe270. The C-6 methyl group of the oleandropyranosyl sugar also made four alkyl contacts with Phe270, Leu267, Phe254 and Val253 of Mcl-1 (Fig. 9b). The ring of the oleandropyranosyl sugar made Pi-alkyl interactions with Val253 and Met250, while the A ring of the aglycon made a Pi-alkyl contact with Arg263 of Mcl-1 (Fig. 9b). Iloneoside was also found to be anchored in the BH3 binding domain of Bfl-1 and Bcl-A1 APB2Ps (Fig. 10). The 3-hydroxyl unit of the allopyranosyl sugar of iloneoside made a sulphur-x contact with Cys55 of Bfl-1. The 5-methyl group on the allopyranosyl sugar also made two alkyl contacts with Lys77 and Val74, while the 5-methyl group on the oleandropyranosyl sugar made three alkyl contacts with Phe95, Leu52 and Val74 of Bfl-1 (Fig. 10(i)). The A ring of the aglycon made a pi-alkyl contact with Val48 of Bfl-1. In the case of Bcl-A1, two tigloyls on the aglycon formed two H-bonds with Lys77. The 3-O-glycosydic bond between the oleandropyranosyl sugar and the 17β-marsdenin aglycon formed a H-bond with Thr91, while the oleandropyranosyl sugar interacted with Gly87 and Val44 in a H-bond and alkyl contact, respectively (Fig. 10(ii)).

**Table 3:** Top docked compounds interaction with the amino acid residues of anti-apoptotic BCL-2 proteins

Compounds	Target Proteins	Hydrophobic interactions	H-Bonding
Navitoclax	Bcl-2	Phe101 Asp108Val153 Arg143 Tyr105 Tyr199 Phe109 Met112 Met112 Leu134	Gly142
Iloneoside		Arg143 Phe101 Ala97 Ala146 Phe195 Tyr199 Met112 Val145 Leu134 Phe150	Gly142
WEHI_539	BCL-XL	Lue108 Phe105 Tyr195 Asp108 Ala149 Arg139 Ala142 Lue130 Arg102 Phe109 Tyr101	Asn136
Iloneoside		Phe97 Tyr101 Phe105 Arg139	Ser106 Gly138
2-Indole-acylsulfonamide		Val249 Leu325 Leu246 Phe270 Ile294 Met250 Phe228 Met231 Thr266 Ala227 His224	Leu267 Arg268 Asn260 Gly262
Gambogic Acid	Mcl-1	His224 Leu246 Leu235 Phe270 Phe228 Met250 Met231 Ala227 Val274 Val249	Thr266 Arg263
Iloneoside		Val253 Phe254 Leu267 Met250 Phe270 Leu235 Leu246 Arg263	Lue246 Val258 Arg263 Gly257
Iloneoside	Bfl-1	EU^52^ VAL^44^ VAL^48^ Lys77 Phe95 Val74 Cys55	
	Bcl-A1	Val44 Arg88	Lys77 Thr91 Gly87

#### Result for molecular dynamics

**Fig. 11:**
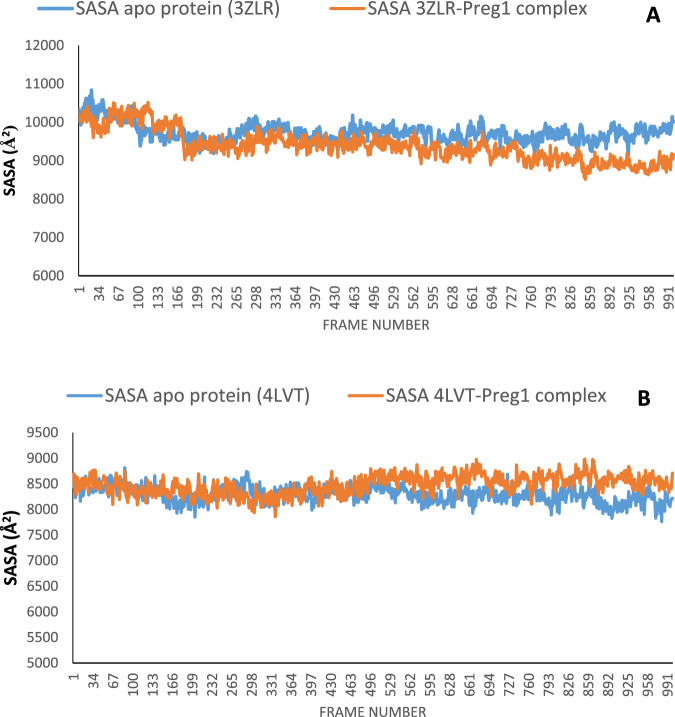
The anti-apoptotic BCL-2 proteins Surface Accessible Surface Area (SASA) plots of molecular dynamics (MD) simulation (**A**) BCL-2-Iloneoside complex (**B**). BCL-XL_Iloneoside complex. Pale orange line: complexes; blue line: apo proteins

**Fig. 12:**
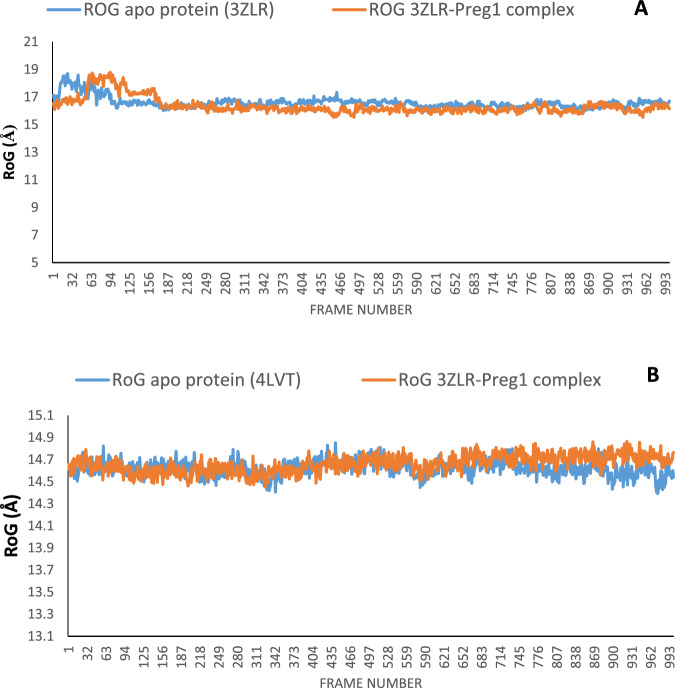
The Radius of gyration (RoG) plots of molecular dynamics (MD) simulation of anti-apoptotic BCL-2 proteins (**A**) BCL-XL_Iloneoside complex (**B**) BCL-2-Iloneoside complex. Blue line:

**Fig. 13:**
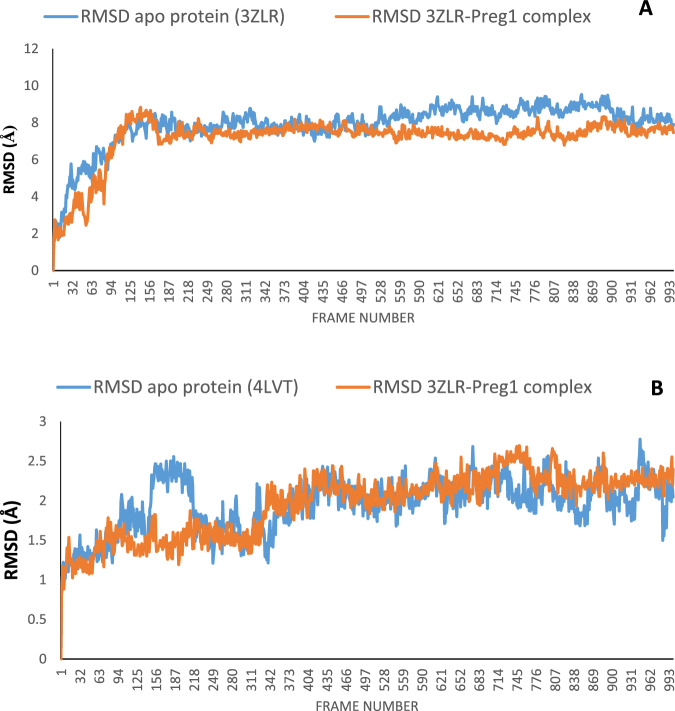
The molecular dynamics (MD) simulation of the anti-apoptotic BCL-2 proteins shows the Backbone-Root Mean Square Deviation (RMSD) plots (**A**) BCL-2-Iloneoside complex (**B**). BCL-XL_Iloneoside complex

**Fig. 14:**
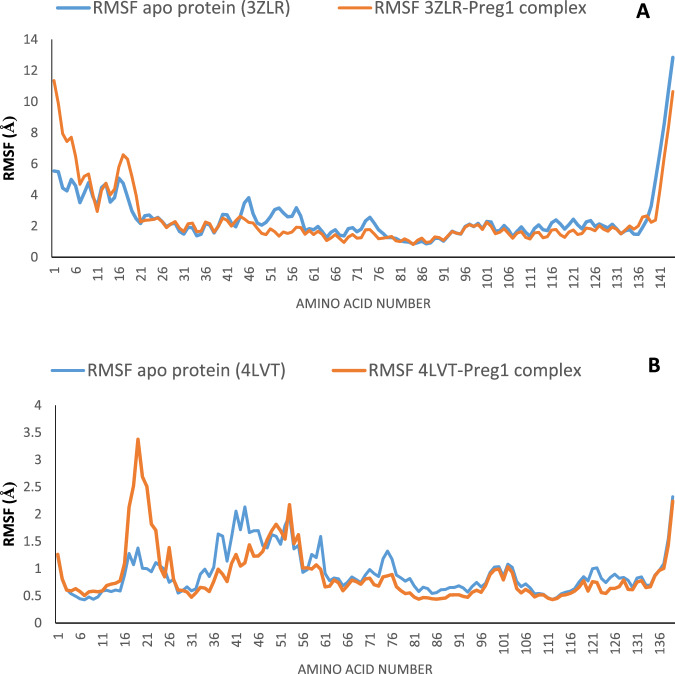
Root Mean Square Fluctuations (RMSF) plots per residue of anti-apoptotic BCL-2 protein molecular dynamics (MD) simulation (**A**) BCL-XL-Iloneoside complex (**B**) BCL-2-Iloneoside complex

**Fig. 15:**
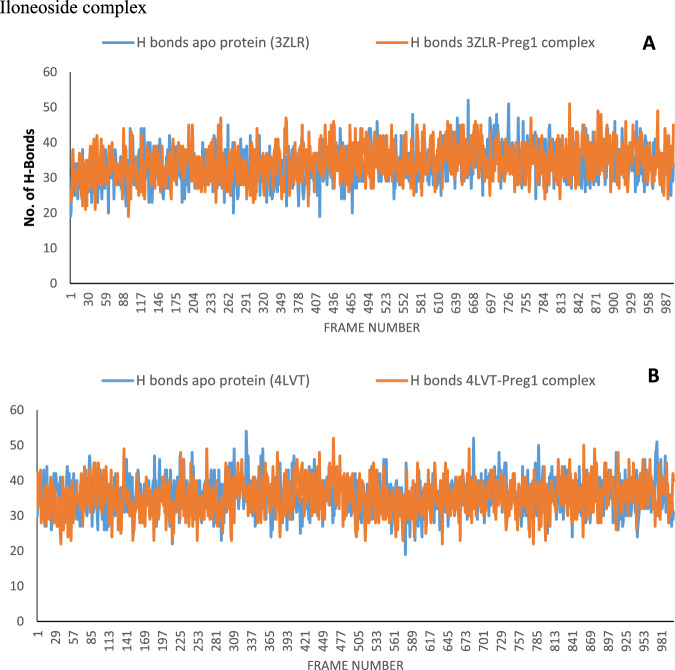
The changes in the number of H-bonds during the MDS trajectory of anti-apoptotic BCL-2 proteins (**A**) BCL-XL-Iloneoside complex (**B**) BCL-2*-*Iloneoside complex. Blue line: Apo protein*s and* Pale orange line: Complexes

The structural fluctuations and stability that occurred in the selected APB2Ps (Bcl-2 and BCL-XL) in the unbound state and in the complexed state with iloneoside were monitored in a full atomic 100 ns simulated molecular dynamic (MD) environment. VMD Tk terminal scripts were used to examine and compute the MDS trajectories. The following thermodynamic parameters were computed: H-bonds, RMSD, RMSF, RoG and SASA. The SASA and RoG values give an indication of the changes in protein folding. The SASA (Fig. 11A) and RoG (Fig. 12A) plots of the Apo protein (unbound) BCL-XL show an initial overall decrease, which is consistent with the rotation of amino acids (S1:G18) by ~180 degrees and their motion towards the rest of the protein, resulting in a more compact form. In contrast, the BCL-XL–iloneoside complex exhibits nearly the same trend as the unbound protein, with an initial short increase followed by a decrease, with average values of 9726 Å^2^ and 9392 Å^2^ for unbound and BCL-XL-iloneoside, respectively. For the BCL-2 protein, the SASA plots show a steady fluctuation around mean values of 8292 Å^2^ and 8480 Å^2^ for the unbound and BCL-2-iloneoside, respectively, indicating no conformational changes along the trajectories. However, the unbound protein shows a decrease from 14 ns to 24 ns, which corresponds to the motion of the amino acids (A41:Q59) close to the rest of the protein, closing the pocket. The RoG plots show a steady fluctuation with a mean of 14.62 Å and 14.65 Å for the unbound and complexed systems, respectively (Figs. 11B and 12B). The RMSD plots show how much each frame has deviated from the initial structure. Comparing the RMSD plots between the apo and BCL-XL-iloneoside complexes shows that both systems follow the same trend. They were equilibrated at around 15 ns with mean values of 8.25 Å and 7.5 Å, respectively (Fig. 13A). The BCL-2 (unbound) system shows fluctuation that corresponds to the same time period in the RoG plots, but the BCL-2–iloneoside system was more stable due to the presence of iloneoside in the binding pocket (Fig. 13B). The RMSF plots (Fig. 14) have spikes both at the C- and N-terminals, which correspond to their rapid fluctuation. In the case of BCL-XL-iloneoside, the spike in the complex at amino acid number 25 corresponds to the motion of a loop that forms an alpha-helix in the apo protein. The average number of H bonds (Fig. 15A) for the BCL-XL and BCL-XL-iloneoside systems is nearly constant, having 33 and 34 H-bonds, respectively, while that of the BCL-2 systems had mean H bonds of 36 and 35, for the apo protein and BCL2-iloneoside system, respectively (Fig. 15B).

#### MM/PBSA calculation

**Fig. 16:**
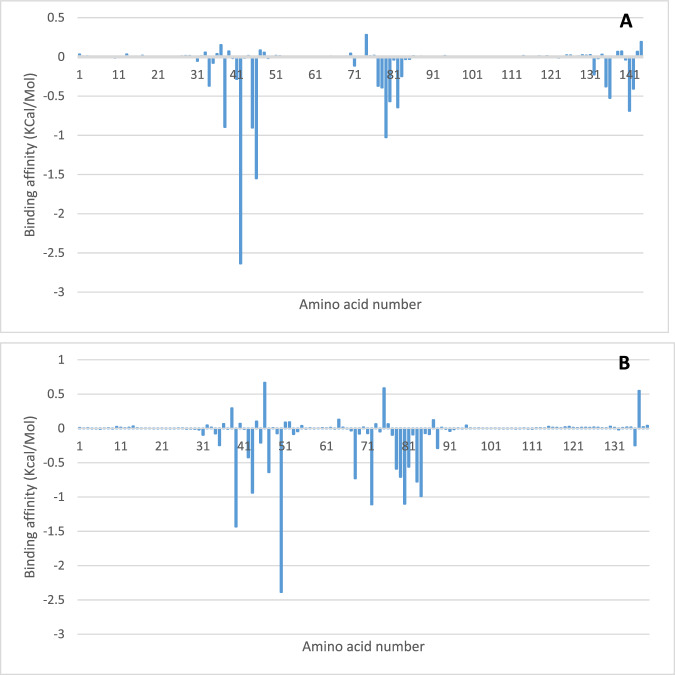
Plot showing binding free energy contribution per residue for the (**A**) BCL-XL and (**B**) BCL-2 iloneoside complexes using molecular mechanics and Poisson-Boltzmann surface area (MM/PBSA)

Binding free energy was calculated using MMGBSA, and the decomposition of the binding free energy was obtained. Figure 16 shows the decomposition of the free binding energy as contributed by the interacting amino acid to iloneoside. The binding free energies of iloneoside to BCL-XL and BCL-2 are −25.72 ± 7.22 and −27.76 ± 11.32 Kcal/mol. The decomposition plot revealed residues Phe38, Tyr42, Ala45, Phe46, Gly79 and Ala141 of BCL-XL to actively contribute to the total free energy (Fig. 16A), while the BCL-2-iloneoside complex was stabilised by Phe39, Tyr43, Phe47, Met50, Val68, Lys72, Asn78, Trp79, Gly80, Arg81, Val83 and Ala84 (Fig. 16B).

#### Clustering analyses of molecular dynamics simulation trajectories

**Fig. 17:**
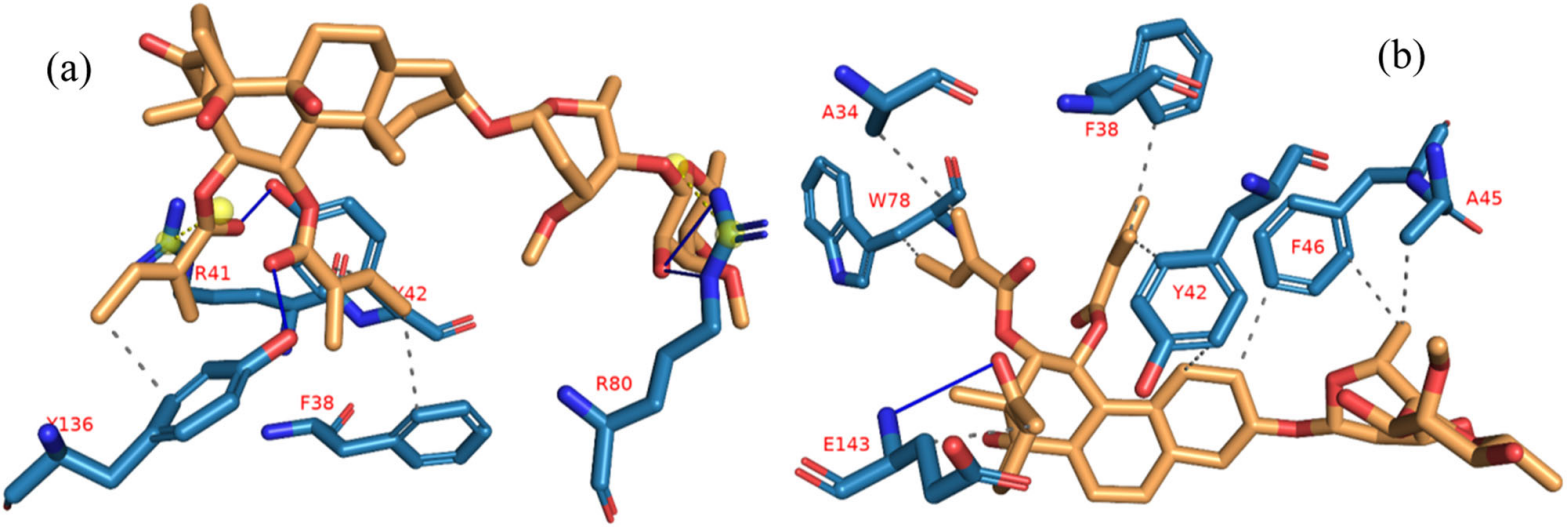
Human BCL-XL-Iloneoside complex interactions in (**a**) cluster 1 and (**b**) cluster 5. Dashed lines with a grey dot indicate hydrophobic interactions. Line: Hydrogen bond; blue, solid. Amino acids are blue molecules represented by a single letter. Luteolin is represented as an orange stick

**Fig. 18:**
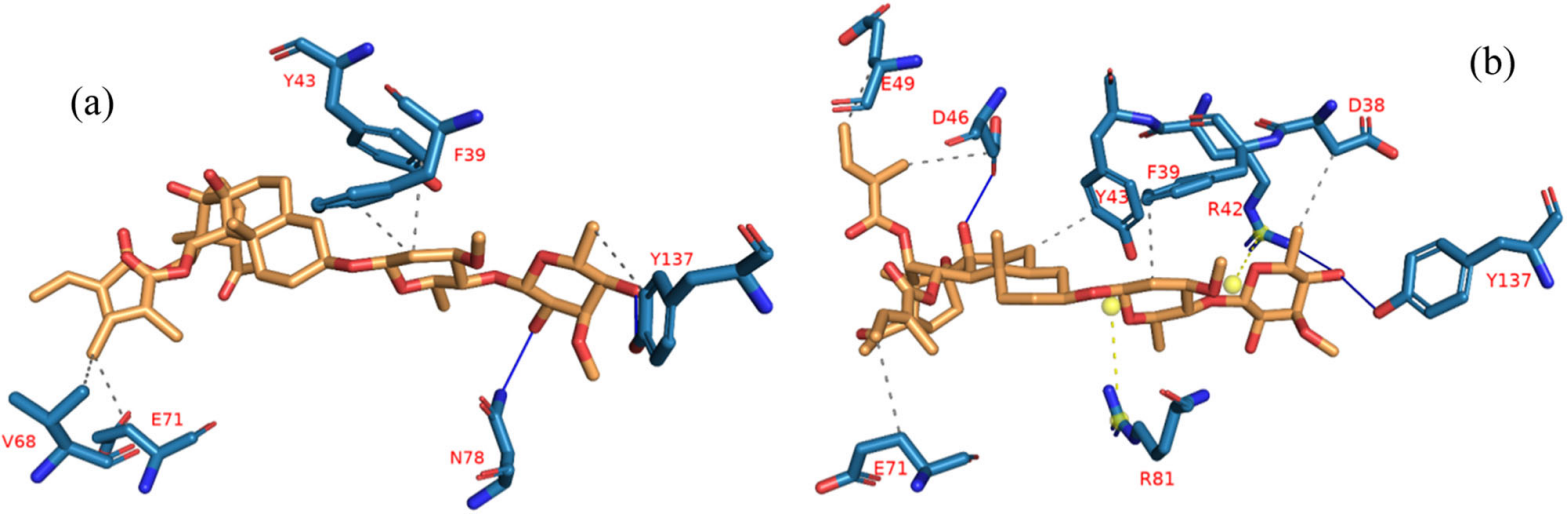
Human BCL-2-Iloneoside acid complex interactions in (**a**) cluster 1 and (**b**) cluster 4. Grey dashed lines indicate connections that are hydrophobic; blue solid lines indicate hydrogen bonds. Amino acids are blue molecules represented by a single letter. The depiction of ursolic acid is an orange stick

The results of the clustering analysis of the MDS trajectories of the BCL-XL-iloneoside and BCL-2-iloneoside complexes are presented in Table 4. Using a representative conformer, the number of clusters that were created as well as the interactions that occurred in various clusters are also displayed. In the complexes, hydrophobic interactions with minimal hydrogen bonds are prevalent. Tyr42 is the most frequently occurring amino acid involved in BCL-XL-iloneoside complex interactions. All of the clusters retrieved from the MDS trajectory retained interactions with these residues, however, Asp46 in BCL-2-iloneoside was retained in the clusters. The interactive view of the first and last clusters in the BCL-XL-iloneoside and BCL-2-iloneoside complexes, respectively, is displayed in Figs. 17 and 18.

**Table 4:** The interactions of iloneoside to different clustered conformation from the cluster analysis of APB2P molecular dynamics simulation (MDS) trajectories

COMPLEX NAME	CLUSTER NUMBER	NUMBER OF INTERACTIONS	HYDROPHOBIC	NUMBER OF INTERACTIONS	HYDROGEN BONDS	NUMBER OF INTERACTIONS	SALT BRIDGES
BCL-XL -Iloneoside	Cluster 1	4	Phe38 **Tyr42 (2)** Tyr136	4	**Tyr42** Arg80 (2) Tyr136	0	None
	Cluster 2	9	Phe38 **Tyr42 (3)** Leua45 Phe46 (2) Val82 Tyr136	2	**Tyr42 Tyr** 136	0	None
	Cluster 3	7	**Tyr42 (2)** Ala45 Phe46 Val82 Tyr136 Ala141	4	**Tyr42** Tyr136 Ala142 (2)	0	None
	Cluster 4	4	Phe38 **Tyr42** Ala45 Val82	0	None	0	None
	Cluster 5	9	Ala34 Phe38 **Tyr42 (2)** Ala45 Phe46 (2) Trp78 Glu143	1	Glu143		
BCL-2- Iloneoside	Cluster 1	5	Phe39 Tyr43 Val68 Glu71 Tyr137	2	Asp78 Tyr137	0	None
	Cluster 2	1	Tyr43	2	**Asp46 (2)**	0	None
	Cluster 3	5	**Asp46** Phe47 Glu49 Val68 Glu71	3	**Asp46** G80 Tyr137	1	Arg45
	Cluster 4	6	Asp38 Phe39 Tyr43 **Asp46** Glu 49 Glu71	4	Arg42 **Asp46 (2)** Tyr137		

The most common amino acids are in **bold**.

Amino acids were numbered from 1 to 144 and 1 to 139 for 3ZLR and 4LVT proteins respectively. The numbers here differ from that of the deposited crystal structure by 59 and 62 respectively.

## Discussion

The cytotoxicity and viability test is an elementary but first line probe into the cytotoxicity screening of several potential anticancer agents. Regardless of the type of cell-based assay being used, the activity of a particular compound or xenobiotic and their growth inhibitory potential can be studied at the cellular level [46]. In our previous study, we reported the growth inhibitory activities of the methanol extract, EtAC fraction, and isolated pregnanes from *G. latifolium* using the CCK-8 cell proliferation assay and Hoechst 33342 staining for morphological examination. In that study, iloneoside was the most potent cytotoxic pregnane against HL-60 cells. The induction of apoptotic morphology in HL-60 cells upon treatment with iloneoside was the likely proposed mechanism [21]. Iweala et al. [47] had previously reported the growth inhibitory effect of the dichloromethane extract of *Gongronema l*. leaf using the MTT cell proliferation assay on human lung carcinoma and human breast adenocarcinoma [22]. Thus far, there is no report of the antiproliferative activities of the extract or its constituents on prostate cancer cells. Consequently, the antiproliferative effect of the extract and the most potent pregnane (iloneoside) isolated in our previous study was examined against prostate cancer cell lines using MTT cell proliferation assay, apoptosis quantification, cell cycle analysis using flow cytometry and computational analysis including ensemble-based molecular docking, molecular dynamics simulation, binding free energy calculation and MD trajectory cluster analysis.

Chemical fingerprinting of phytocompounds from extracts and formulations has been a delineating link between the chemical constituent and the bioactivity of the whole [48]. UPLC-ESI-TOFMS provides a fast and extensive identification probe that confers a unique fingerprint on the ubiquitously distributed phytochemicals in plant samples. Recently, there has been some profiling of known plants with the aim of keeping a database of their constituents and relating this to their medicinal potentials [49–51]. he chromatographic profile of our previously isolated compounds **1–5** [21] in the EtOAc fraction revealed the abundance of compounds **1–4** as major constituents of the EtOAc fraction, while **5** was present in a trace amount. The various retention times for the compounds, which are a reflection of their polarity, provide useful information that is critical for the preservation of this plant and its secondary metabolites, as this has never been reported before [52]. Meeting the same conditions as the stationary and mobile phases, the phytochemicals can be isolated directly from the ethyl acetate fraction. The marked difference in the retention time of compound **1** compared to those of compounds **2**, **3** and **4** is the presence of an additional tigloyl substituent at C-11 and C-12. This might have increased the polarity of the compound, thereby increasing the retention time. Engstrom et al. [53] used this method to analyse over 120 extracts, produced from as little as 1 mg of plant powder, in a day. This provided an easy-to-use, comprehensive and rapid addition to the medicinal chemistry tools currently employed for the qualitative and quantitative screenings.

For the in vitro cytotoxicity studies, two different strains of human prostate carcinoma (PC3 and DU145) cell lines with distinctive features were treated with the extract and iloneoside. Though both PC3 and DU145 human prostate carcinoma are known not to express androgen receptors and prostate specific antigen (PSA), PC3 is reported to be more metastatic when compared to DU145 [54]. From the antiproliferative analysis, a significant concentration-dependent inhibition (p ≤ 0.05) on the selected strains of prostate cancer cells was observed (at two different time points, 24 and 48 h). It was observed that the extract of *G. latifolium* exerted a strong growth inhibition against the proliferation of PC3 and DU145, as assessed by the IC_50_ via MTT assay. The IC_50_ was lower when compared to the effect on HL-60 cells, lung carcinoma and human breast adenocarcinoma cell lines [47]. Iloneoside, the most active constituent in our previous study against HL-60 cells, exhibited low inhibitory activities against PC3 and DU145. The apoptosis quantification and cell cycle analysis using flow cytometry further showed that the iloneoside induced apoptosis in a few cells at the highest dose of 200 uM.

With more molecular biological data available, the application of computer-based analysis of molecular interactions between possible therapeutic options and target receptors has improved [55]. The identification of compounds with potential applications as bioactive chemicals has been accomplished through the integration of computational investigations and experimental studies. By simulating the atomic-level interaction between a small molecule and a protein using the molecular docking technique, we may clarify basic biochemical processes and describe the behaviour of small molecules in target proteins’ binding sites [56]. During a docking study, a critical protein in a pathway may also be targeted to determine which ligand can bind strongly to the protein’s active site, thereby offering inhibitory function to the function of the protein [57].

Herein, we performed an in-depth simulation-based molecular docking analysis, often referred to as ensemble-based docking analysis [58], against five apoptotic protein targets.

Results from our ensemble-based molecular docking to the d BCL-XL and BCL-2 APB2P show that iloneoside was docked into the hydrophobic BH3 binding grove in some close resemblance with a selective BCL-XL co-crystalized ligand (WEHI 539). Iloneoside interacted with the conserved aspartate on the BH3 domain and the conserved Arg139 in the BCL-XL groove. A previous report established that the interactions in two P2 and P4 hydrophobic pockets are responsible for the high binding affinity of peptides to both BCL-2 and BCL-XL. The hydrophobic interactions of iloneosides within P2 were buttressed by a hydrogen bond to the carbonyl backbone of Ser106; this interaction was overserved by the binding of WEHI 539 to the BCL-XL groove. Iloneoside also interacted with the guanidinium moiety of Arg139. Iloneoside, just like navitoclax, did not form any direct electrostatic interactions with the conserved arginine of BCL-2. This is in contrast with the binding of WEHI 539 to BCL-XL. Also, the MCL-1 BH3 domain is defined by a hydrophobic core including Val253, Val249, Ala227 and Leu267 [32].

From the MD simulation trajectories that were generated for both the apo and the complex system, several thermodynamic parameters (RMSD, RMSF, RoG, SAS and H-bonds number) were computed. The RMSD is a credible measure for protein stability. The close RMSD mean values of the apo and iloneoside bond complexes and corresponding RMSD plots showed that the binding of iloneoside did not distort the integral structure of the BCL-XL and BCL-2 APB2P [59]. The flexibility of different regions of BCL-XL and BCL-2 APB2P and the amino acid residues along the trajectory were measured using the RMSF plots. Residues with higher RMSF values connote greater fluctuations. The close RMSF values for the apo and iloneoside-bound BCL-XL and BCL-2 APB2P show that the binding of iloneoside did not distort the conformation integrity of the protein [59, 60]. The SASA values and the plots showed that the binding of iloneoside did not change the compacted nature of the APB2P. This fact was further confirmed by the RoG plots, which show that the APB2P did not undergo any unfolding in the accessible surface area for the protein. The close average number of hydrogen bonds shows that the intramolecular hydrogen bonds in BCL-XL and BCL-2 were not disturbed by binding of the APB2P [61].

Furthermore, results from simulation-based molecular binding quantification have furnished more accurate estimates of ligand binding energies, which are often referred to as binding free energies [62]. In these calculations, the free energy difference between the complex and the unbound states of proteins and the free ligands is employed, thus providing in-depth information about the binding modes of the hits in drug design [63]. Furthermore, the decomposition of the total binding free energy into its respective quantities provides insights into the contributing amino acid residue. The decomposition of the MM-GBSA binding free energy for contributing amino acids further collaborated the static docking calculation. Clustering of trajectories separates the MDS conformations into structurally homogeneous groups, this gives a quick understanding of the interactions during the process of simulation [64]. Analysis of all the clusters showed that the interactions with important residues were maintained throughout the simulation.

## Conclusion

Herein, we investigated the antiproliferative effect of the *Gongronema latilolium* extract and the most active pregnane (iloneoside) that was previously isolated from our previous study against prostate cancer cell lines using the MTT cell proliferation assay, apoptosis quantification, cell cycle analysis using flow cytometry, and computational analysis. The extract demonstrated significant concentration dependent inhibition (p ≤ 0.05) on the proliferation of both PC3 and DU145 prostate cancer cells at both 24 and 48 h. Iloneoside, the most active constituent in our previous study against HL-60 cells, exhibited low inhibitory activities against PC3 and DU145. Although iloneoside, presented low inhibitory activities, apoptosis quantification and cell cycle analysis using flow cytometry further showed that iloneoside induced apoptosis in a few cells at a dose of 200 uM. The ensemble-based molecular docking of the iloneoside and marsectohexol to the BCL-XL and BCL-2 APB2P, shows that the iloneoside was tightly docked into the hydrophobic BH3 binding grove in some close resemblance with the co-crystalized ligand, where they interact with amino acid residues that are implicated in the inhibition of the activities of the proteins. The MMGBSA binding free energy contribution per amino acid residue revealed interaction with interaction with catalytic residues. The MDs analysis showed that iloneoside was stable during the 100 ns MDs analysis, while the clustering of the trajectories obtained from the MDs showed that the interactions were strongly preserved even in a dynamic environment. Using apoptotic quantification, ensemble-based docking and MDs, we have been able to extend our previous study on the apoptotic mechanism of iloneoside. Together, the results show that *Gongronema latilolium* has antiproliferative compounds with iloneoside, in part, or synergy with other constituents may be responsible for the antiproliferative activities of the leaf, subject to further investigation.
